# Answering biological questions by analysis of the strawberry metabolome

**DOI:** 10.1007/s11306-018-1441-x

**Published:** 2018-10-26

**Authors:** Annika Haugeneder, Johanna Trinkl, Katja Härtl, Thomas Hoffmann, James William Allwood, Wilfried Schwab

**Affiliations:** 1 Biotechnology of Natural Products, Technische Universität München, Liesel-Beckmann-Str. 1, 85354 Freising, Germany; 2 Environmental and Biochemical Sciences Group, The James Hutton Institute, Invergowrie, Dundee, Scotland DD2 5DA UK

**Keywords:** Strawberry, Fragaria, Metabolite, Volatile, VOC, Aroma, Metabolomics, Volatilomics

## Abstract

**Background:**

The qualitative and quantitative analysis of all low molecular weight metabolites within a biological sample, known as the metabolome, provides powerful insights into their roles in biological systems and processes. The study of all the chemical structures, concentrations, and interactions of the thousands of metabolites is called metabolomics. However present state of the art methods and equipment can only analyse a small portion of the numerous, structurally diverse groups of chemical substances found in biological samples, especially with respect to samples of plant origin with their huge diversity of secondary metabolites. Nevertheless, metabolite profiling and fingerprinting techniques have been applied to the analysis of the strawberry metabolome since their early beginnings.

**Aim:**

The application of metabolomics and metabolite profiling approaches within strawberry research was last reviewed in 2011. Here, we aim to summarize the latest results from research of the strawberry metabolome since its last review with a special emphasis on studies that address specific biological questions.

**Key scientific concepts:**

Analysis of strawberry, and other fruits, requires a plethora of analytical methods and approaches encompassing the analysis of primary and secondary metabolites, as well as capturing and quantifying volatile compounds that are related to aroma as well as fruit development, function and plant-to-plant communication. The success and longevity of metabolite and volatile profiling approaches in fruit breeding relies upon the ability of the approach to uncover biologically meaningful insights. The key concepts that must be addressed and are reviewed include: gene function analysis and genotype comparison, analysis of environmental effects and plant protection, screening for bioactive compounds for food and non-food uses, fruit development and physiology as well as fruit sensorial quality. In future, the results will facilitate fruit breeding due to the identification of metabolic QTLs and candidate genes for fruit quality and consumer preference.

## Introduction

In addition to biopolymers including proteins, polysaccharides, lignins, and deoxynucleic acids, organisms ingest and produce a multitude of low-molecular weight molecules collectively called the metabolome (Oliver et al. 1998). In plants, the small molecules determine key features such as nutritional value, flavour, fragrance, colour, bioactivity, pest and disease antagonism. The definition of metabolome was coined in analogy to the previously suggested notions genome and proteome although the difficulty with the concept was immediately recognized, as molecules making up the metabolome are more diverse in their physiochemical properties than structures comprising the genome and proteome. The metabolome embraces a wide range of structurally and chemically various molecules including highly water-soluble and volatile compounds such as short-chain alcohols and fruit esters, respectively, but also completely water-insoluble and non-volatile metabolites like carotenoids and monosaccharides, respectively. Due to the high complexity, until today there is no analytical instrument and method available to detect, identify and quantify all of the metabolites that constitute the metabolome. It is also impossible to determine or even estimate the number of different chemicals contributing to the metabolome of a sample. This will only be feasible when single molecule analysis becomes a reality. Amylose, although a biopolymer, may serve here as an example, which is formed by the successive addition of d-glucose units to a growing saccharide chain. As amylose contains 200–2000 monosaccharide units, all intermediates could theoretically be present in a sample and thus amylose alone comprises already several hundreds of metabolites. In other words, metabolomics, which is the systematic study of all metabolites in a given biological sample, is unrealistic considering the current analytical equipment and methods. The high-throughput characterization of selected groups of small molecules presently performed in many labs is best described as metabolic profiling when the identities of the metabolites are known (targeted analysis) and metabolic fingerprinting when the exact chemical structures of the compounds are unknown (untargeted analysis). The traditional target molecules analysis aims at the detection and quantification of a small set of known metabolites but it is not applicable for the chemical profiling of complex mixtures.

As analysis of the metabolome can link key features of interest with the causal compounds, numerous life science disciplines currently apply metabolome analysis techniques to tackle biological questions. In plant science, metabolite profiling of soft fruits is of special interest because low molecular weight compounds are essential constituents of a healthy diet due to their potential health-beneficial bioactivity and fast and comprehensive analysis of their metabolite composition can significantly accelerate the breeding of nutrient-rich varieties. Hundreds of natural products, including primary and secondary metabolites, have been found in fruit but it is expected that many more are present, which will be identified in the near future due to continuous improvements in the sensitivity and separation efficiency of the analytical instruments.

**Fig. 1:**
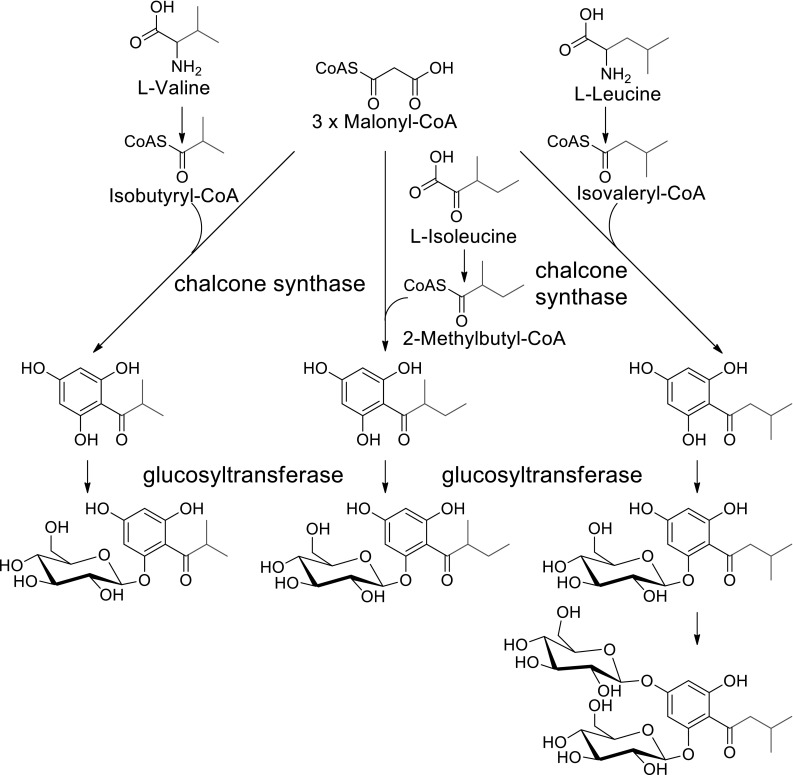
Biosynthetic pathway of acylphloroglucinols newly identified in strawberry fruit (*F. x. ananassa*) (Song et al. 2015a, 2016a)

The only review on the strawberry (*Fragaria x ananassa*) metabolome dates back to 2011 (Hanhineva et al. 2011). In their excellent report, Hanhineva and co-workers discussed the non-volatile phytochemicals produced in leaf, flower and fruit of the strawberry plant, both with regard to the physiology of the plant and as a component of the human diet. They presented the metabolome of strawberry in connection with developments and applications of cutting-edge analytical chemistry-based approaches. Since then, the list of clearly identified strawberry metabolites has not been extended very much, except for acylphloroglucinols (Song et al. 2015b, 2016b) but the analysis of the metabolome has been employed to investigate various biological issues. Acylphloroglucinols are newly identified strawberry fruit constituents and are formed by the condensation of intermediates in branched-chain amino acid metabolism with three molecules of malonyl-CoA catalysed by chalcone synthase (CHS) (Song et al. 2015b) (Fig. 1). Identification of acylphloroglucinols was made possible by comparative metabolite fingerprinting analysis of fruits of wild type and transgenic plants showing reduced *CHS* transcript levels.

Thus, this review cannot present newly identified strawberry compounds, except for acylphloroglucinols but focuses and discusses recent successful examples applying metabolite profiling and fingerprinting analysis to study strawberry plant physiology with the aim to improve fruit quality. The focus is on the analysis of secondary metabolites because they have been best studied in strawberry fruit and there are no studies, which focus only on the metabolite profiling of primary metabolites in strawberry fruit.

## Primary and secondary metabolites

Although biopolymers such as proteins, polysaccharides, lignins, and deoxyribonucleic acids are major constituents of plant cells they are not considered as part of the cells metabolome. Generally, the metabolome refers to small-molecule chemicals (< 1500 Da, arbitrarily defined) found in biological samples, which can be easily analysed by high-throughput platforms based on GC–MS and LC–MS equipment (Wishart 2007). In contrast to the analysis of small molecules, the comprehensive analysis of polymers cannot be achieved so easily in high throughput and automated applications because of a dearth of methods that are available for profiling these complex high-molecular weight biomolecules.

The metabolome itself is further subdivided into primary and secondary metabolites, which are either directly involved in the normal growth of the organism or have important ecological function, respectively. Because secondary metabolism uses biosynthetic enzymes and building blocks derived from primary metabolism, both are directly connected. Primary metabolism comprises all physiological processes allowing organisms to grow and propagate while the secondary or specialized metabolism is essential for an organisms’ interaction with the environment. Although not necessary for survival, specialized metabolites aid a plant in protection, competition and species interactions. The substances of the primary and secondary metabolism are considered as the chemical phenotype, which can be investigated by metabolite profiling and metabolite fingerprinting analyses.

Phenylpropanoids (mainly glucose esters of cinnamic acid, 4-coumaric acid, caffeic acid and ferulic acid), flavonoids (mainly glycosides of quercetin and kaempferol as well as derivatives of catechin and epicatechin), anthocyanins (mainly glycosides of pelargonidin and cyanidin), and ellagic acid derivatives are among the major non-volatile secondary metabolites found in strawberry fruit (Hanhineva et al. 2011). They are involved, among others, in the attraction of frugivores (anthocyanins), protect plants against UV-B light (flavonoids), serve as energy-rich precursor molecules in plant metabolism (phenylpropanoid glucose esters), and play a role in plant growth regulation (derivatives of ellagic acid). Recent studies have shown that these compounds contribute to the nutritional quality of foods due to their anti-inflammatory, photoprotective, anticarcinogenic and antimutagenic properties (Giampieri et al. 2014).

In addition to non-volatile organic chemicals, plants also produce and emit volatile metabolites. The volatile substances (volatilome) comprise a structurally diverse group of low molecular weight chemicals having an appreciable vapour pressure under ambient conditions. They attract pollinator and seed dispersing animals, provide defence against pathogens and pests, and play a role in plant–plant communication. Only recently, the volatilome of the strawberry fruit has been excellently reviewed (Ulrich et al. 2018) and in total 979 volatiles were identified in strawberry fruit. Despite the fact that only a small number of the volatile metabolites contributes to the aroma of strawberry, the knowledge is important for genetic studies, plant breeding, plant protection, nutritional science, and the strawberry processing industry.

## Biological questions

### Gene function analysis and genotype comparison

**Fig. 2:**
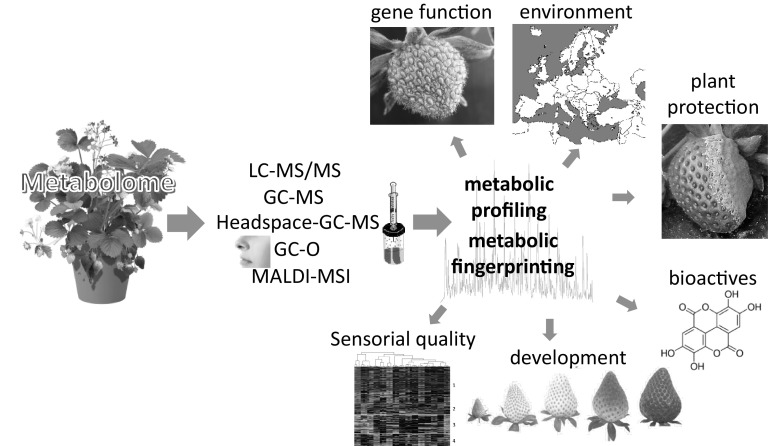
Functional analysis of the strawberry metabolome

Metabolome analysis is a powerful tool for studying gene function in organisms and has been frequently applied to elucidate the roles of nucleic acids in strawberry fruit development (Fig. 2). Generally, in reverse genetics, the gene’s function is altered and the effect on the phenotype, including the perturbations of the chemical phenotype is analysed. Already in 2006, RNAi-induced gene silencing in strawberry fruit by agroinfiltration was demonstrated as a rapid assay for gene function analysis (Hoffmann et al. 2006). Later, the efficiency was experimentally confirmed by deep sequencing of the primary and secondary siRNAs (Härtl et al. 2017b). Successful applications of this method have been reviewed in 2011 (Schwab et al. 2011), 2015 (Guidarelli and Baraldi 2015) and 2016 (Carvalho et al. 2016).

Transient RNAi-mediated downregulation of transcript levels in strawberry fruit in combination with metabolome analysis is now commonly utilized for studying gene function during fruit development and ripening, e.g., for the validation of genes potentially involved in polyphenol and flavour biosynthesis in strawberry fruit. Among others, the transcription factor *MYB10* (Medina-Puche et al. 2014), the *anthocyanidin reductase* gene (Fischer et al. 2014), *glycosyltransferase* genes (Song et al. 2015a), genes involved in the biosynthesis of polyphenols (Ring et al. 2013), acylphloroglucinols (Song et al. 2015b, 2016b), and 4-hydroxy-2,5-dimethyl-3(2H)-furanone glucoside (Song et al. 2016a) were functionally characterized by metabolite profiling analysis of polyphenols in reverse genetics studies.

Furthermore, comparative metabolite profiling analysis of ripe fruits of different *Fragaria* species (Muñoz et al. 2011) and of red- and white-fruited strawberry varieties (Härtl et al. 2017a) were performed in combination with expression profiling of regulatory and biosynthetic genes (Hossain et al. 2018) to gain a deeper insight into the regulation of anthocyanin biosynthesis. Chemical analysis by LC–MS of anthocyanins, flavonols, flavan-3-ols, flavanones, hydroxycinnamic acid derivatives, and ellagic acid in fruits of a *F. vesca* near isogenic line (NIL) collection revealed that the genetic factor was more decisive than the environmental factor for the accumulation of phenolic compounds (Urrutia et al. 2016). Genotyping the NIL collection allowed the mapping of 76 stable quantitative trait loci controlling accumulation of the phenolics.

Strawberry is an economically important fruit crop worldwide and there is a growing need to have analytical methodologies available for the authentication of variety and origin. Metabolite profiling technologies have revealed significant differences between three strawberry cultivars and thus provides a suitable tool to differentiate strawberry cultivars and their production regions (Akhatou et al. 2016). Similarly, unique phenolics were identified in fruits of diploid *Fragaria vesca* compared to octoploid *Fragaria x ananassa* providing the basis for comparative analysis of polyphenols in different strawberry genotypes (Sun et al. 2014).

### Environmental effects

Environmental stresses are features of life that affect all organisms, in particular plants that have a sessile life style. Metabolite profiling tools are widely applied to explore stress metabolism and to elucidate its regulation in a wide range of environmental conditions and biological systems. Thus, the influence of different biotic and abiotic environmental factors on the metabolite profiles has also been frequently analysed in *Fragaria*.

A comparative analysis of secondary metabolites of *Fragaria x ananassa* fruits obtained from either organic or conventional, non-organic crops was performed (D’Urso et al. 2015). Although an untargeted metabolite fingerprinting approach differentiated both sample sets, flavonoids and anthocyanins were not responsible for the classification. Similarly, the untargeted and targeted analysis of the phytochemical content of wild and cultivated *Fragaria vesca* fruit collected from different geographic areas revealed that fruits from locations with different pedoclimatic conditions could be discerned (D’Urso et al. 2016). Chemical analysis also revealed significant changes in primary and secondary metabolites between three strawberry cultivars grown under different crop conditions (Akhatou et al. 2016, 2017).

In another study, 186 secondary metabolites of strawberry fruits were separated by targeted metabolic profiling analysis showing significantly different concentrations in 15 strawberry cultivars grown in Finland or Estonia (Kårlund et al. 2016). Besides, identifying cultivar-specific differences in the levels of major phytochemicals, the research also suggested that cultivar selection is essential for breeding strawberry cultivars with optimal functional properties and sensory qualities.

To study the metabolic processes and regulatory mechanisms during cold exposure of strawberry, metabolite profiling approaches were applied to both root and leaf tissues (Koehler et al. 2015; Rohloff et al. 2012). About 160 substances comprising primary and secondary metabolites were quantitatively analysed. Metabolic changes included a strong adaptation of the central metabolism, and accumulation of osmotically active sugars, amino acids, and amines. The amino acid proline, known to be cold induced in other plant systems, was absent. Levels of key metabolites of the cold-inducible raffinose pathway, such as galactinol and raffinose were drastically enhanced. The results showed that alterations in metabolite pools of cold-acclimated *Fragaria vesca* were clearly affected by the genotype. Overall, the cold response behaviour of the strawberry plant is characteristic for a moderately cold tolerant plant.

In nine strawberry genotypes grown at various altitudes the influence of temperature, ultraviolet (UV)-irradiation and sunshine duration on the phytochemical composition of fruits was investigated by metabolite profiling analysis (Palmieri et al. 2017). A significant correlation was found between the levels of benzoic acid derivatives and altitude as well as phenylpropanoid content and UV-radiation and sunshine duration. The flavone concentrations seemed to be affected by the variety, while flavonol and ellagitannin levels were highly controlled by the environment. The accumulation of numerous secondary metabolites reflects the acclimation effects of plants in response to abiotic stress.

### Plant protection

Polyphenols are major constituents in strawberry leaves and potentially possess defensive activities against microbial pathogens. The levels of defensive polyphenols can be increased by exposing plants to elicitor-active compounds. Treatment of strawberry leaves with the plant activator S-methylbenzo-1,2,3-thiadiazole-7-carbothiate, followed by metabolite profiling analysis resulted in increased levels of individual ellagitannins, while treatment with a birch wood distillate elicitor strongly enhanced the concentrations of chlorogenic acid in comparison with the non-treated control (Kårlund et al. 2014). The outcome suggested that plant elicitors are useful tools for the activation of defensive metabolites in strawberry leaves.

In another study, the metabolic response of strawberry leaves to the angular leaf spot bacterium (*Xanthomonas fragariae*) was examined by metabolite profiling analysis (Kim et al. 2016). Infected leaves showed decreased levels of selected molecules of the phenylpropanoid pathway, which are related to the plant defence system. In contrast, infection of strawberry fruit with *Colletotrichum nymphaeae*, the causative agent of strawberry black spot, resulted in the accumulation of high levels of total sugars and increased the contents of ellagic acid derivatives, flavonols, oligomeric procyanidins, flavan-3-ols, and total phenolics (Mikulic-Petkovsek et al. 2013).

Immature strawberry fruits are known to be more tolerant to diseases, the two major strawberry fungal pathogens, *Botrytis cinerea* and *Colletotrichum acutatum* show disease symptoms only at red ripe stages of fruit development (Nagpala et al. 2016). Metabolite profiling analysis revealed pathogen-specific alterations of the phenolic content only in white infected fruits. Interestingly, changes in the expression levels of polyphenol pathway genes did not correlate with the alterations in phenolic content.

Infection of strawberry fruits by *Botrytis cinerea* resulted in a characteristic earthy, mushroom odour caused by 1-octen-3-one and 1-octen-3-ol. Headspace GC–MS techniques were used to determine the alterations in the composition of volatiles as a function of the degree of *Botrytis cinerea* infection, which was determined by an enzyme-linked immunosorbent assay (Vandendriessche et al. 2012). Because some infected fruits showed no symptoms and did not produce fungal volatiles it was concluded that the analytical techniques are not currently useful for early detection of *Botrytis cinerea* infections in strawberry fruit.

### Screening for bioactive compounds for food and non-food uses

The vegetative parts of the *Fragaria* sp. plant are of pharmaceutical interest in many areas of the world and are considered as a promising bioresource for diverse health-related applications. Decoctions of leaves are used for hypertension treatment and show diuretic and dermatological protective properties. Metabolite profiling analysis in strawberry leaves in combination with antioxidant measurement was performed to identify natural products that are accountable for the antioxidant activity of the extracts (D’Urso et al. 2018). LC–MS was also utilized to study the differences and commonalities between the metabolite profiles in leaves (Kårlund et al. 2017). In general, strawberry leaves contained high levels of quercetin and kaempferol derivatives, caffeic and chlorogenic acid derivatives, and ellagitannins as well as octadecatrienoic acid derivatives.

In another study, the strawberry fruit metabolome was analysed in combination with bioactivity tests to identify the key constituent exerting anti-inflammatory activity (Lee et al. 2014). It was found that ellagic acid is the major component playing a crucial role in inflammation in murine macrophage cells. The results demonstrated that metabolite profiling analysis is a versatile tool to determine the key ingredients having biological functions in plant extracts. Additional LC–MS analyses showed that strawberry extracts contain very complex mixtures of ellagitannins and proanthocyanidins, whereas profiles of extracts of raspberry and cloudberry present similar although lower complexity mixtures of ellagitannins and ellagic acid (McDougall et al. 2008).

Comparative LC–MS analyses of extracts from fruits of two Chilean white strawberry genotypes (*Fragaria chiloensis* spp. *chiloensis* and spp. *patagonica*) and of the commercial strawberry *Fragaria x ananassa* (Simirgiotis et al. 2009), as well as of fruits, rhizomes and leaves of *Fragaria chiloensis* were performed (Simirgiotis and Schmeda-Hirschmann 2010). Procyanidins, ellagitannins, ellagic acid and flavonol derivatives were the major products and the different extracts exhibited similar antioxidant activity. Thus, the rhizomes and leaves are also rich sources of phenolic antioxidants.

Selenium (Se) is an essential nutrient for humans, whereas its role in plants remains to be demonstrated. Nevertheless, plant growth is enhanced if Se is added to the cultivation substrate. Metabolite analyses of Se-fortified strawberry fruit revealed an increase in the levels of flavonoids and polyphenols, and an increase in the sweetness of the fruits upon Se accumulation (Mimmo et al. 2017). Strawberry plants appear to be a good target for Se biofortification, as they would increase the human intake of this essential micronutrient.

### Fruit development and physiology

The quality of strawberry fruit is a function of the levels of metabolites, which are affected by different factors, such as the genotype and various environmental impacts, as has been shown before, but also result from physiological alterations during fruit development and ripening (Hanhineva et al. 2011). By applying a range of metabolite profiling technologies in combination with metabolite correlation and network analyses, it has been shown in larger fruit varieties, namely cantaloupe melon that spatial metabolite distributions throughout different fruit tissue sections (epicarp, outer, middle and inner mesocarp) change significantly. These changes become more pronounced during fruit ripening, especially with regard to the ripening of fruit in the anoxic environment of the inner mesocarp. Here, hypoxia has profound effects on both aroma related volatile compounds and their small molecule precursors, as well as leading to the accumulation of ethanol (Biais et al. 2009, 2010; Moing et al. 2011). With technological advancements, especially with respect to technologies capable of studying spatial metabolite distributions (i.e., mass spectrometric (MS) imaging), as well as enhanced cell sorting and micro-dissection techniques, similarly powerful studies will become possible to study the spatial development and ripening in smaller soft fruit species.

Although there are crucial limitations associated with MS imaging such as the inability to differentiate isobaric metabolites, Matrix-Assisted Laser Desorption/Ionization Mass spectrometric Imaging (MALDI–MSI) is the most widely used method to spatially localize metabolites in their native histological context. A number of strategies have been recently proposed to attenuate the disadvantages including in-tissue chemical derivatization and dual isotope labelling of precursor metabolites (Feldberg et al. 2018).

MALDI–MSI was already employed to analyse the spatial distribution of strawberry metabolites during fruit development in one wild-type and two transgenic lines (Crecelius et al. 2017). The investigated metabolites were exclusively present in the skin of the mature fruits and fruit ripening led to a reduction of many of the studied plant constituents. In another study, pelargonidins were detected in the skin, cortical, and pith tissues, whereas cyanidins and delphinidins were found in the skin of the receptacle (Enomoto et al. 2018). Additionally, cyanidins were detected in the skin of the achenes. Citric acid was localized in cortical tissue while hexoses were found almost equally throughout the fruits.

In addition histochemical screening combined with metabolite profiling analysis demonstrated flavan-3-ols (catechins) as secondary metabolites in roots of Rosaceae (Hoffmann et al. 2012). Thus, catechin, epicatechin and dimeric flavan-proanthocyanidins are prominent polyphenols in root tips of strawberry, apple, rose, pear and plum roots and probably play a role as plant protective agents.

Metabolite profiling and fingerprinting analyses in combination with metabolite correlation and network analysis in strawberry has already revealed interdependencies of individual plant constituents and metabolic pathways (Zhang et al. 2011). The outcome of such analyses have provided new insights into strawberry fruit composition and metabolite changes and demonstrated the value of metabolome analysis as a tool for functional genomics.

### Fruit sensorial quality: search for odour-active metabolites and candidate genes

Fruit colour and flavour are important traits of strawberry varieties as they are the main quality parameters for the consumption of strawberry fruits. Thus, many efforts have been undertaken to identify genes controlling fruit colour but also flavour (Schwab et al. 2008). In one report, two cultivars, varying for presence-absence of the volatile γ-decalactone, along with a segregating population, were analysed by metabolite profiling and RNAseq (Chambers et al. 2014). Computational analysis revealed a candidate gene that was likely controlling this key flavour molecule in strawberry.

Gas chromatography with olfactometry (GC/O) can be considered as an untargeted analysis method to differentiate between odour-active and odour-neutral substances of complex blends. The sensitive “nose” detector recognizes substances, which escape detection by other methods, due to their low abundance or instability. GC/O has been employed to examine strawberry fruits harvested in winter months in Florida with short photoperiods to examine the aroma profiles of strawberries grown in subtropical regions (Du et al. 2011). Twenty-nine odour-active volatiles were identified and the cultivars showed an aroma pattern different from those strawberry fruits produced in summer months, probably due to a lack of methyl anthranilate.

GC/O in combination with GC–MS was also used for the identification of volatiles contributing to the aroma of *Fragaria chiloensis* fruit (Prat et al. 2014). This genotype is endemic to Chile, produces white fruits and is one of the progenitors of *Fragaria x ananassa* Duch. Twenty major volatiles were found that showed dilution factors greater than 1000 and thus are associated with the aroma of the Chilean strawberry.

Fruit volatiles were also analysed by GC–MS in a *Fragaria vesca*/*Fragaria bucharica* near isogenic line collection to investigate fruit quality traits (Urrutia et al. 2017). The study revealed 50 major quantitative trait loci including 14 key volatiles, with the transcriptome analysis suggesting new candidate genes for the regulation of the volatile QTLs.

The strawberry volatilome has been very recently reviewed (Ulrich et al. 2018). In total, 979 volatile organic compounds were identified in 25 significant analytical studies between 1997 and 2016, but none of the identified volatiles were consistently reported across all of the studies reviewed. The extraction method was identified as the crucial step that restricted inter-laboratory comparability, as has also been observed in tomato and other species of fruit such as melon (Allwood et al. 2014).

Breeding of new strawberry varieties aims at the improvement of strawberry fruit quality including colour, texture taste and aroma. Biochemically diverse strawberry fruit were subjected to metabolite profiling and consumer rating to identify fruit attributes influencing hedonics and sensory perception (Schwieterman et al. 2014). Consumer preference of strawberry fruit was greatly affected by sweetness and flavour intensity. Thirty-one volatiles were identified which significantly correlated to flavour intensity.

## Conclusions

Metabolomics is the comprehensive large-scale chemical analysis of the metabolome. Due to the chemical complexity of the metabolome and limitations of the instrumental methodologies, a comprehensive and complete analysis of all metabolites is not currently possible. However, alterations in the metabolome are frequently explored by profiling approaches to study biological processes. In studies aimed at the improvement of strawberry fruit quality, combinations of different metabolite profiling techniques have been successfully applied to elucidate gene functions, analyse environmental effects, screen for bioactive compounds, improve plant protection, localize metabolites, compare genotypes and physiological stages and identify odour-active volatiles. The levels of biological insight captured within such plant metabolite profiling studies, demonstrates the great utility of profiling the metabolome and volatilome, when such approaches are combined with other ‘omic’ level data (i.e., transcripts, proteins, enzymatic determinations of metabolite concentrations). The level of systematic knowledge that can be gained is huge especially in contrast with applications within other scientific fields that solely aim to discover biomarker compounds.
