# Correction to: Answering biological questions by analysis of the strawberry metabolome

**DOI:** 10.1007/s11306-019-1480-y

**Published:** 2019-03-09

**Authors:** Annika Haugeneder, Johanna Trinkl, Katja Härtl, Thomas Hoffmann, James William Allwood, Wilfried Schwab

**Affiliations:** 1 Biotechnology of Natural Products, Technische Universität München, Liesel-Beckmann-Str. 1, 85354 Freising, Germany; 2 Environmental and Biochemical Sciences Group, The James Hutton Institute, Invergowrie, Dundee, Scotland DD2 5DA UK

## Correction to: Metabolomics (2018) 14:145 10.1007/s11306-018-1441-x

The article Answering biological questions by analysis of the strawberry metabolome, written by Annika Haugeneder, Johanna Trinkl, Katja Härtl, Thomas Hoffman, James William Allwood and Wilfred Schwab, was originally published electronically on the publisher’s internet portal (currently SpringerLink) on 26 October, 2018 without open access. After publication in volume 14. Issue 11, Citation Id 145, with the author(s)’ decision to opt for Open Choice the copyright of the article changed on 20 December, 2018 to © The Author(s) [2019] and the article is forthwith distributed under the terms of the Creative Commons Attribution 4.0 International License (http://creativecommons.org/licenses/by/4.0/), which permits use, duplication, adaptation, distribution and reproduction in any medium or format, as long as you give appropriate credit to the original author(s) and the source, provide a link to the Creative Commons license and indicate if changes were made.

The original article has been corrected.

